# Efferocytosis by bone marrow mesenchymal stromal cells disrupts osteoblastic differentiation via mitochondrial remodeling

**DOI:** 10.1038/s41419-023-05931-9

**Published:** 2023-07-14

**Authors:** Emily R. Quarato, Noah A. Salama, Allison J. Li, Charles O. Smith, Jane Zhang, Yuko Kawano, Matthew McArthur, Jane L. Liesveld, Michael W. Becker, Michael R. Elliott, Roman A. Eliseev, Laura M. Calvi

**Affiliations:** 1 Department of Environmental Medicine, University of Rochester Medical Center, Rochester, NY USA; 2 James P. Wilmot Cancer Institute, University of Rochester Medical Center, Rochester, NY USA; 3 Center for Musculoskeletal Research, University of Rochester Medical Center, Rochester, NY USA; 4 Department of Microbiology and Immunology, University of Rochester Medical Center, Rochester, NY USA; 5 Department of Internal Medicine, Yale School of Medicine, New Haven, CT USA; 6 Department of Medicine, University of Rochester Medical Center, Rochester, NY USA; 7 University of Virginia, Department of Microbiology, Immunology, and Cancer Biology, Charlottesville, VA USA; 8 Department of Orthopedics, University of Rochester Medical Center, Rochester, NY USA; 9 Department of Pathology and Laboratory Medicine, University of Rochester Medical Center, Rochester, NY USA

**Keywords:** Cell death and immune response, Bone, Bone marrow cells, Stem-cell differentiation

## Abstract

The efficient clearance of dead and dying cells, efferocytosis, is critical to maintain tissue homeostasis. In the bone marrow microenvironment (BMME), this role is primarily fulfilled by professional bone marrow macrophages, but recent work has shown that mesenchymal stromal cells (MSCs) act as a non-professional phagocyte within the BMME. However, little is known about the mechanism and impact of efferocytosis on MSCs and on their function. To investigate, we performed flow cytometric analysis of neutrophil uptake by ST2 cells, a murine bone marrow-derived stromal cell line, and in murine primary bone marrow-derived stromal cells. Transcriptional analysis showed that MSCs possess the necessary receptors and internal processing machinery to conduct efferocytosis, with Axl and Tyro3 serving as the main receptors, while MerTK was not expressed. Moreover, the expression of these receptors was modulated by efferocytic behavior, regardless of apoptotic target. MSCs derived from human bone marrow also demonstrated efferocytic behavior, showing that MSC efferocytosis is conserved. In all MSCs, efferocytosis impaired osteoblastic differentiation. Transcriptional analysis and functional assays identified downregulation in MSC mitochondrial function upon efferocytosis. Experimentally, efferocytosis induced mitochondrial fission in MSCs. Pharmacologic inhibition of mitochondrial fission in MSCs not only decreased efferocytic activity but also rescued osteoblastic differentiation, demonstrating that efferocytosis-mediated mitochondrial remodeling plays a critical role in regulating MSC differentiation. This work describes a novel function of MSCs as non-professional phagocytes within the BMME and demonstrates that efferocytosis by MSCs plays a key role in directing mitochondrial remodeling and MSC differentiation. Efferocytosis by MSCs may therefore be a novel mechanism of dysfunction and senescence. Since our data in human MSCs show that MSC efferocytosis is conserved, the consequences of MSC efferocytosis may impact the behavior of these cells in the human skeleton, including bone marrow remodeling and bone loss in the setting of aging, cancer and other diseases.

## Introduction

Maintenance of homeostasis in the bone marrow microenvironment (BMME) depends on efficient clearance of dead and dying cells [1, 2]. In the bone marrow, this role is primarily fulfilled by bone marrow macrophages [3], which serve as professional phagocytes. The phagocytic clearance of apoptotic cells, also termed “efferocytosis”, is an essential process for tissue homeostasis [1, 2, 4, 5]. The different types of cellular death (apoptosis, necrosis, ferroptosis, pyroptosis) can confer pro-inflammatory or anti-inflammatory signaling in part through modulation of efferocytosis [6, 7]. While efferocytosis is a specialized form of phagocytosis, and the molecular mechanisms of efferocytosis closely resemble those of phagocytosis, the outcomes are different. For instance, phagocytosis induces inflammation and antigen presentation, whereas efferocytosis is typically immunologically silent [8, 9]. Apoptotic cells expose and secrete signals, known as the “find-me signals”, to attract phagocytes, and “eat-me signals”, to promote engulfment and then a return to homeostasis, also known as resolution [10, 11]. Many organs and systems have supporting, non-professional phagocytes that can be recruited to aid in the clearance of dead and dying cells and inflammation [3, 4, 12–15]. A handful of studies have shown that mesenchymal stromal cells (MSCs) are capable of both phagocytosis and efferocytosis and may play the role of the non-professional phagocyte within the BMME [16–26]. Under normal physiologic conditions, MSCs give rise to bone-forming osteoblasts and are critical for maintaining skeletal homeostasis. However, little is known about the impact efferocytic MSCs play in the BMME and on skeletal homeostasis.

In macrophages, efficient and repetitive efferocytosis requires mitochondrial remodeling, specifically a shift toward mitochondrial fission [27, 28]. Mitochondria are dynamic organelles central to cellular energy production, metabolite synthesis, redox balance, epigenetic and apoptotic signaling, and ion homeostasis that promote growth and maturation in nearly every cell type [29–33]. Mitochondrial dynamics are also important regulators of MSC differentiation. Notably, dysfunction of homeostatic metabolism and mitochondrial remodeling have been found to alter osteoblastic differentiation and bone formation [34–42]. During osteoblastic differentiation of MSCs, mitochondrial networks exhibit increased fusion, likely to better meet the energy demands of osteoblastic differentiation and matrix production [35]. Since efferocytosis leads to mitochondrial fission and MSC differentiation requires mitochondrial fusion, efferocytosis by MSCs may represent a previously unidentified mechanism of MSC dysfunction and impaired osteoblastic differentiation. Here, we demonstrate that efferocytosis by MSCs leads to mitochondrial fission and inhibits osteoblastic differentiation.

## Methods

### In vitro efferocytosis assays

ST2 cells, a bone marrow derived mesenchymal stromal cell line, were a kind gift of Dr. Clifford Rosen (Maine Medical Research Center). ST2 cells were plated at 2 × 10^4^ per cm^2^ in αMEM without ascorbic acid (Gibco)+10% FBS + 1% pen-strep and incubated in 21% oxygen at 37 °C until 80% confluency. Neutrophils were isolated from bone marrow of young (8–12 weeks) C57BL/6 mice using the EasySep^TM^ Mouse Neutrophil Enrichment Kit (Stem Cell Technologies) as we previously published [43] and incubated in RPMI + 10% FBS + 10 mM HEPES overnight (18–20 h) at 37 °C/5%CO_2_ to force cells to become end-stage (a.k.a. exhausted) neutrophils as previously described [44]. End-stage neutrophils were washed with PBS and fluorescently labeled with 20 nM efluro670 (ThermoFischer) according to manufacturer’s instructions. Targets (neutrophils or apoptotic lymphocytes) were given in excess (1 MSC:10 Target) to plated MSCs and incubated for up to 24 h. Cells were then washed 3× with PBS, imaged, and collected for flow cytometry analysis.

### Confocal microscopy

ST2 cells were infected with a lentivirus to ubiquitously express mCherry. The pLVX-EF1a-IRES-mCherry vector (Clontech) contains an EF1a promoter to constitutively express IRES-mCherry in the infected cells. The detailed methods for generating lentiviral particles, and infecting cells are described as previously published [45]. The construct was co-transfected with pPax2 (provides packaging proteins) and pMD2.G (provides vesicular stomatitis virus-g envelope protein) plasmids into 293TN (System Bioscience) cells to produce lentiviral particles that were used to infect ST2 cell lines. After expanding the cells after infection, mCherry-positive ST2 cells were sorted on FACS Aria cell sorter (BD Bioscience) for subsequent experiments. Following successful infection, cells were plated at 2 × 10^4^ per cm^2^ in ascorbic acid-free αMEM + 10% FBS + 1% pen-strep and incubated at 21% O_2_/5% CO_2_ at 37 °C until 80% confluent. Neutrophils were isolated from bone marrow of young (8–12 weeks) C57BL/6 UBC-GFP mice using the EasySep^TM^ Mouse Neutrophil Enrichment Kit (Stem Cell Technologies) and incubated in RPMI + 10% FBS + 10 mM HEPES overnight (18–20 h) at 37 °C/5%CO_2_ as previously described [44]. End stage neutrophils were then given at a 1:1 ratio to plated MSCs in hypoxia (5% O_2_/5% CO_2_) at 37 °C for 24 h. Cells were visualized using an inverted Nikon Ti2-E microscope at room temperature using an air-plan apochromat VC x20/0.75 objective. NIS-Elements C with JOBS Acquisition Module software was used to acquire and analyze all images. Work was supported by the Wilmot Cancer Center Imaging and Radiation Shared Resource.

### RNA sequencing of murine MSCs

Following sacrifice, soft tissue was removed from the bilateral tibiae, femora, and pelvic bones and bones were each cut into 3–4 pieces. Bone pieces were crushed with a mortar and pestle to release bone marrow (BM) into PBS + 2%FBS. Bone marrow was passed through a 16 G needle to disassociate clumps and pelleted by centrifugation of 1200 *RPM* for 5 min. Red blood cells were removed via incubation in RBC lysis buffer (156 mM NH_4_Cl, 127 μM EDTA, and 12 mM NaHC_3_) for 5 min. BM was digested in HBSS containing collagenase type IV (1 mg/mL; Sigma), dispase (1 mg/mL, Gibco), and DNase (10 units/mL, New England Biolabs) for 35 min at 37 °C. Digested BM was filtered through a 100 µM cell strainer (Corning) and washed with PBS + 2%FBS. Cell numbers were determined using the TC20 Automated Cell Counter (Biorad) and Trypan Blue (Sigma-Aldrich) to exclude dead cells. A two-step approach was used to remove hematopoietic cells, first via magnetic-depletion and second via fluorescence-activated cell sorting (FACS). For magnetic-depletion of hematopoietic populations, BM was labeled with biotinylated antibodies against CD45 and lineage markers (Ter119, B220, CD3e, and Gr1) followed by secondary labeling with streptavidin-conjugated magnetic particles (IMag Streptavidin Particles Plus-DM, BD Biosciences). BM was incubated on the BD IMagnet to magnetically separate CD45+ and lineage+ hematopoietic cells from the non-hematopoietic fraction enriched for stromal cells. The stromal cell-enriched fraction was then labeled with PE-CF594 streptavidin, PerCP-Cy5.5 lineage antibodies, APC-Cy7 CD45, FITC CD31, and PE CD51. Cells were labeled with DAPI to exclude dead cells and FACS-purified using a FACSAria II (BD Biosciences) to remove residual hematopoietic cells (lineage+ and/or CD45+) and endothelial cells (CD31+) to obtain lineage- CD45− CD31− CD51+ marrow stromal cells. Sorted marrow stromal cells were seeded in 12-well plates at 1000 cells/cm^2^ in αMEM (ascorbic acid-free) +10%FBS + 1%P/S and incubated in 2%O_2_/5%CO_2_/37 °C. Media was changed on day 4 of culture initiation and every 3–4 days thereafter. Upon reaching confluence, cells were passaged and expanded in 6-well plates. For passaging, cultures were washed with PBS and treated with TrypLE Express (ThermoFisher Scientific) to detach cells. Equivalent volume of culture media was added, and cells were re-plated at ratios ranging from 1:5 to 1:10. Marrow stromal cells were used at passage 2 or 3 for experiments. In all stromal cultures, flow cytometry was used to assess expression of hematopoietic and macrophage markers (lineage, CD45, CD11b, F4/80), endothelial markers (CD31), and stromal markers (CD51, Sca1, CD140a) and confirmed lack of contamination with macrophages and endothelial cells (data not shown).

Marrow stromal cultures were grown in 6-well plates prepared as described above. Each well was pre-treated with 1 mL of media containing 5 × 10^6^ apoptotic thymocytes/well. Primary murine apoptotic thymocytes were isolated and prepared as previously published [46], fluorescently labeled with 20 nM efluro670 (ThermFischer) according to manufacturer’s instructions. Culture plates were centrifuged for 40 s at 100 × *g* and incubated in 5%O_2_/5%CO_2_/37 °C for 24 h. Control cultures received no target. Stromal cells were washed 3–6× with PBS to remove non-engulfed phagocytic targets. Treated and control cells were then washed 3× with PBS and then collected by FACS-isolated directly in RLT Plus buffer (Qiagen). Both populations were sorted and control as well as target+ cells were isolated. RNA extraction was performed with Qiagen RNeasy PLUS Micro kit following standard operating procedures of the URMC Genomic Core. RNA quality was assessed using Agilent Bioanalyzer 2100. One nanogram of high-quality (RNA integrity number >8.0) total RNA from each sample was reverse-transcribed into cDNA using the Clontech SMART-Seq v4 Ultra Low Input RNA Kit. Final Illumina libraries were constructed using 200 ng of cDNA with the Illumina 2500HiSeq Library Preparation Kit. All data was analyzed using R version 4.1.0 with benjamini-hochberg corrections and packages for DESeq2(version 1.32.0) with LFC shrinkage software ashr (version 2.2-47), GO.db (version 3.13.0), and EnrichR (version 3.0) with a gene alignment set KEGG_2019_mouse. GEO dataset can be accessed at https://www.ncbi.nlm.nih.gov/projects/geo/query/acc.cgi?acc=GSE223283. Pathway analysis was performed separately on upregulated and downregulated significantly differentially expressed genes (DEGs) with an adjP < 0.05, a baseMean cutoff > 100 read counts, and no log fold change cutoff. Gene set enrichment analysis (GSEA) was performed on all genes above a base Mean cutoff > 100 read counts without curation for individual gene significance, direction, or fold change.

### RNA sequencing of ST2 cells

ST2 cells were plated at 2 × 10^4^ per cm^2^ in αMEM without ascorbic acid (Gibco)+ 10% FBS + 1% pen-strep and incubated in normoxia at 37 °C until 80% confluent. Neutrophils were isolated from human peripheral blood via Mono-Poly resolving medium (MP Biomedicals, Inc) according to manufacturer’s instructions and incubated at −80 °C in FBS + 10% DMSO for a minimum of 18 h. End-stage neutrophils were washed with PBS and fluorescently labeled with 20 μM eFluor670 dye (eBioscience) in PBS at 37 °C for 20 min and then washed with RPMI + 10% FBS + 10 mM HEPES to bind free dye. End-stage neutrophils were then given in excess (10:1) to plated MSCs for 3 or 24 h. Cells were then washed 3× with PBS and collected for isolation via sorting flow cytometry analysis (BD FACSAriaII). ST2 cells and isolated neutrophils were FACS-isolated directly in RLT Plus buffer (Qiagen). RNA extraction was performed with Qiagen RNeasy PLUS Micro kit following standard operating procedures of the URMC Genomic Core. RNA quality was assessed using Agilent Bioanalyzer 2100. One nanogram of high-quality (RNA integrity number >8.0) total RNA from each sample was reverse-transcribed into cDNA using the Clontech SMART-Seq v4 Ultra Low Input RNA Kit. Final Illumina libraries were constructed using 150 pg of cDNA with the Illumina Nextera XT DNA Library Preparation Kit. Differential gene expression was analyzed using R version 4.1.0 using DESeq2(version 1.32.0) with benjamini-hochberg correction and LFC shrinkage software ashr (version 2.2-47). Gene set enrichment (Kolmogorov-Smirnov) and pathway analysis (hypergeometric test) were assessed using EnrichR (version 3.0), and ClusterProfiler (version 3.13) with databases GO.db (version 3.13.0), and KEGG_2019_mouse. GEO dataset can be accessed at https://www.ncbi.nlm.nih.gov/projects/geo/query/acc.cgi?acc=GSE223283. Pathway analysis was performed separately on upregulated and downregulated significantly differentially expressed genes (DEGs) with an adjP < 0.05, a baseMean cutoff > 100 read counts, and no log fold change cutoff. Gene set enrichment analysis (GSEA) was performed on all genes above a base Mean cutoff > 100 read counts without curation for individual gene significance, direction, or fold change.

### Bioenergetic profiling

Oxygen consumption rate (OCR) and extracellular acidification rate (ECAR) were measured using Seahorse XF96 (Seahorse Bioscience). Cells were plated on Seahorse 96-well plates 24 h before experiment at a density of 3 × 10^3^ cells/well. Immediately before the experiment, media was replaced with DMEM-XFmedia containing 5 mM glucose, 1 mM glutamine, 1% serum, and no pyruvate. A baseline measurement of OCR and ECAR was taken, and then an inhibitory analysis was performed using injections of oligomycin (Olig) at 1 µM, FCCP at 0.5 µM, and antimycin A (AntA) at 1 µM. After analysis, cells were trypsinized and counted. The following OxPhos and glycolytic indexes were calculated: basal respiration (OCRpre-Olig - OCRpost-AntA), ATP-linked respiration (OCRpre-Olig - OCRpost-Olig), maximal respiration (OCRpost-FCCP - OCRpost-AntA), respiratory capacity (OCRpost-FCCP - OCRpre-Olig), proton leak (OCRpost-Olig - OCRpost-AntA), basic glycolysis (ECARpre-Olig), glycolytic capacity (ECARpost-Olig), and glycolytic reserve (ECARpost-Olig - ECARpre-Olig). ATP was measured using the CellTiter-Glo kit (Roche).

### Measuring mitochondrial networking

Human MSCs (hMSC) cells were seeded on laminin coated 25 mm glass coverslips that are placed inside of wells on a 6-well plate and incubated at 5%O_2_/5%CO_2_/37 °C. hMSCs were exposed to end-stage neutrophils (described above) stained with calcein AM (ThermoFisher) at 1 μM. hMSCs were then incubated in HBSS containing mitochondria-specific fluorescent probe, MitoTracker Red (ThermoFisher), at 100 nM to visualize mitochondria. hMSCs were visualized using the AxioVert microscope and ×40 magnification. Images were captured and analyzed in ImageJ using Mitochondrial Network Analysis (MiNA) plugin for ImageJ. ImageJ Convolve feature was applied allowing for the stained mitochondria to be highlighted. The threshold feature highlights mitochondria, minimizing the background. In the process of analyzing, only mitochondria >25 pixels were selected to further minimize background noise [47].

### Measuring mitochondrial membrane potential

Human MSCs (hMSC) were exposed to end-stage neutrophils (described above) stained with calcein AM (ThermoFisher) at 1 μM. After washing, hMSCs were incubated in phenol red-free media containing membrane potential (${\Delta}{\Psi}\mathrm{m}$) sensitive probe Tetramethylrhodamine ester, TMRE (ThermoFisher), at 20 nM for 30 min at 37 °C. In parallel, a set of cells undergoing efferocytosis was stained with Nonyl Acridine Orange (NAO, ThermoFisher) at 100 nM to detect possible changes in mitochondrial mass. Cells were then lifted from plates and analyzed using 18-color LSR Fortessa flow cytometer (BD Biosciences). DAPI was present in the assay media to gate out dead cells. DAPI^-^ (viable)/calcein^+^ (efferocytic)/TMRE^+^ cells were analyzed for TMRE signal to measure ${\Delta}{\Psi}\mathrm{m}$ in efferocytic cells. As a negative control, cells were added with antimycin A at 1 μM to depolarize mitochondria. A difference in TMRE signal between polarized and depolarized mitochondria was taken as a measure of ${\Delta}{\Psi}\mathrm{m}$. In parallel, DAPI^−^ (viable)/calcein^+^ (efferocytic)/NAO^+^ cells were analyzed for NAO signal to measure mitochondrial mass. TMRE signal was normalized to NAO signal to account for possible changes in mitochondrial mass.

### Differentiation assays

ST2 cells were plated at 2 × 10^4^ per cm^2^ in αMEM without ascorbic acid (Gibco)+10% FBS + 1% pen-strep and incubated in 5%CO_2_/37 °C until 80% confluent. Neutrophils were isolated from bone marrow of young (8–12 weeks) C57BL/6 mice using the EasySep^TM^ Mouse Neutrophil Enrichment Kit (Stem Cell Technologies) and incubated in RPMI + 10% FBS + 10 mM HEPES overnight (18–20 h) at 5%CO_2_/37 °C as previously described [44]. End-stage neutrophils were washed with PBS and fluorescently labeled with 20 nM efluro670 (ThermoFischer) according to manufacturer’s instructions. Targets were then given at baseline (1:1) and in excess (1:2 and 1:3) to plated MSCs for 24 h. Following incubation, cells were washed 3× with PBS, imaged, and given supplemented media every 2–3 days for 21 days to differentiate down osteoblastic (αMEM with ascorbic acid (Gibco) + 10% FBS + 1% pen-strep + 10 mM β-glycerolphosphate + 50 μg/mL ascorbic acid) or adipocytic lineage (α-MEM media without ascorbic acid (Gibco) + 10% FBS + 1% pen-strep + 5000 nM insulin + 100 nM dexamethasone) or sorted for PMN+/− cells alongside controls and replated at 2 × 10^4^ per cm^2^ for 24 h before being given supplemented media as described above. Cells were stained for alkaline phosphatase and von Kossa for mineralization formation or with BODIPY for lipid formation every 7 days for 21 total days.

### BODIPY staining

Cells were washed with PBS 3×, then stained with 10 mM BODIPY (Invitrogen™) used at 1:1000 ratio in media (α-MEM media + 10% FBS + 1% pen-strep) for 30 min at room temperature (RT) in the dark. Following the incubation, the cells were washed 3× with PBS and imaged via light microscopy. Images were then quantified for adipocyte formation using ImageJ software.

### Alkaline phosphatase and Von Kossa staining

Cells were washed with PBS 3× and then fixed with formalin for 30 min at RT. Following fixation, the cells were washed with water 3× and left to sit for 15 min RT while preparing alkaline phosphatase (AP) stain. To prepare the AP stain, 5 mg Naphthol AS MX-PO4 was dissolved in 200 uL of N,N-dimethylformamide (DMF), 25 mL 0.2 M Tris pH8.3 and 25 mL water. Red Violet LB salt (30 mg) was added to solution, vortexed and filter through 45 um filter. After incubation with AP stain for 45 min RT, cells were washed with water 3× and then stained with 2.5% silver nitrate for 30 min RT in ventilated area, then washed with water and air dried. All plates were imaged via light microscopy and quantified for osteoblastic formation using ImageJ software.

### Light microscopy

Images were also taken at room temperature on an Olympus BX41, Olympus DP70 camera, and 20× and 4× UPlanFl objective (NA 0.5). CellSense software (Olympus) was used to acquire images. All images captured in bright field and with filters (FITC) were overlaid using ImageJ software.

### Efferocytosis assays with pharmacological inhibition of mitochondrial fission

Primary human MSCs (hMSCs) cells (Lonza) were plated at 2 × 10^4^ per cm^2^ in αMEM + 10% FBS + 1% pen-strep and incubated in 5%O_2_/5%CO_2_/37 °C until 80% confluent. Neutrophils were isolated from human peripheral blood via Mono-Poly resolving medium (MP Biomedicals, Inc) according to manufacturer’s instructions and incubated in RPMI + 10% FBS + 10 mM HEPES overnight (18–20 h) at 37 °C/5%CO_2_ as previously described [44]. hMSCs were treated with 25 μM Mdivi for 1 h prior to giving 20 nM efluro670 (ThermoFischer) stained end-stage neutrophils in excess (1:10) for 24 h. Cells were then washed 3× with PBS, imaged, and collected for flow cytometry analysis.

### Alkaline phosphatase measurements with pharmacological inhibition of mitochondrial fission

Cells that performed efferocytosis in the presence or absence of Mdivi, were washed and stained for AP as described above. Wells were photographed and images analyzed using ImageJ for staining intensity.

### Flow cytometry

All samples were run on a LSRII flow cytometer: 3 lasers, 355 nm, 488 nm, and 633 nm (BD Biosciences). Analysis was performed using FlowJo version 10.8 (Tree Star). Sorting was done on a FACSAria II with 405-, 488-, 532-, and 633 nm lasers (BD Biosciences). All flow cytometry equipment is housed and quality controlled within the URMC Flow Cytometry core.

### Statistics

All data are presented as mean ± SD. All analyses were performed with GraphPad Prism software (version 9.2.0) using two-tailed Student’s *t* test, 1-way or 2-way ANOVA with Tukey’s multiple-comparisons post-test as appropriate. A *p* value < 0.05 was considered significant, any values nearing significance were stated exactly.

## Results

### Mesenchymal stromal cells participate in efferocytosis

**Fig. 1:**
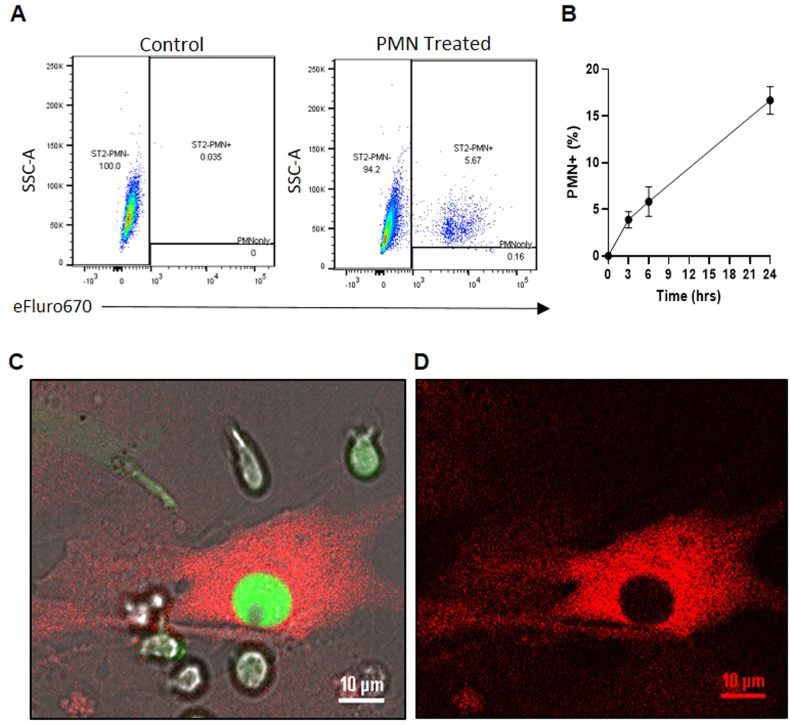
MSCs can conduct efferocytosis. **A**, **B** Representative flow cytometry gating scheme and quantification of ST2 cells incubated with eFluoro670 labelled end stage neutrophils (PMNs) for 24 h. *N* = 6. Mean ± SD shown on graph. **C**, **D** Representative microscopy images showing uptake of end stage neutrophils (GFP+) by ST2 cells (mCherry+) and the resulting void in the cytoplasm. **Videos E**, **F** Confocal microscopy video (middle right) and z-stack captured of ST2 cells up taking end stage neutrophil (GFP+).

Previous studies have noted that MSCs can phagocytose bacteria [16, 17], metallic particles from prosthetics [18, 19, 22], collagen [23], and apoptotic cells [20, 21, 24–26]. However, dynamics and impact of this MSC activity remain poorly understood. Billions of cells return to the bone marrow to be cleared daily by phagocytes, with a large component being neutrophils (up to 60%) [48], making them a likely efferocytic target for professional phagocytes, such as macrophages, and non-professional phagocytes, such as MSCs. To investigate the impact of efferocytosis on MSC, we first performed flow cytometric analysis of neutrophil uptake by ST2 cells, a murine bone marrow derived mesenchymal stromal cell line [49]. The assay showed that ST2 cells conduct efferocytosis of end-stage murine neutrophils (PMNs) (Fig. 1A, B, Supplementary Fig. 1). Through microscopy, we confirmed that ST2 actively engulfed end-stage PMNs, as evidenced by the void left in the cytoplasm and z-stack imaging (Fig. 1C, D, Videos E–F). Taken together, these data confirm that MSCs can actively participate in efferocytosis, however the impact on MSCs ability to support normal function following efferocytosis remains to be elucidated.

**Fig. 2:**
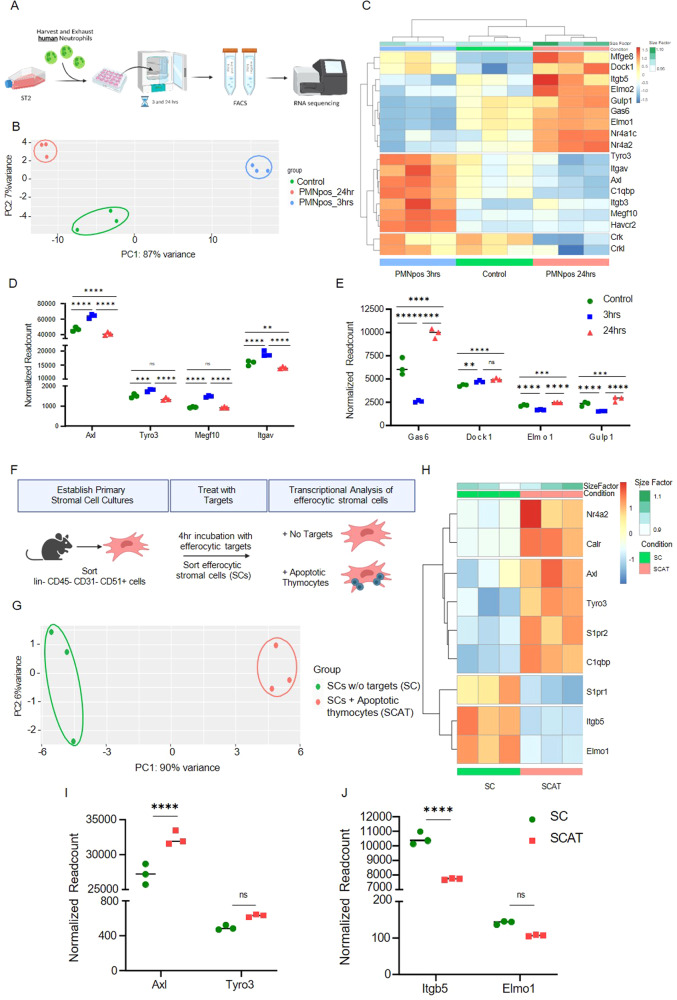
MSCs upregulate efferocytic receptor-pathways following efferocytosis. **A** ST2 experimental setup (**B**) Principal component analysis (PCA) of 3 and 24 h sorted efferocytic ST2 cells via bulk RNA sequencing. **C** Heat map and (**D**, **E**) read count of efferocytic and intracellular processing genes at 3 h or 24 h vs the control analyzed via a DESeq2 with a benjamini-hochberg FDR correction. **F** Schematic representation of primary mMSC experimental setup (**G**) Principal component analysis of 4 h sorted efferocytic mMSCs cells fed apoptotic thymocytes via bulk RNA sequencing. **H** Heat map and (**I**, **J**) Read count of efferocytic and intracellular processing genes at 4 h SCAT vs SC analyzed via a DESeq2 with a benjamini-hochberg FDR correction.

In general, non-professional phagocytes have a limited efferocytic capacity in comparison to professional phagocytes, as they do not possess as many phagocytic receptors or produce reactive oxygen species (ROS) as readily to assist with degradation of internalized targets [4, 50–52]. To define the efferocytic machinery utilized by MSCs, we conducted RNA sequencing on ST2 cells exposed to excess human PMNs (1:10). Cells were then harvested at 3 and 24 h after the addition of PMNs and separated by fluorescence-activated cell sorting (FACS) based on presence of the target label (Fig. 2A, Supplemental Fig. 2). At quality control (QC) check via bioanalyzer, RNA from human PMN targets was highly degraded and gave insufficient RNA quantity and RNA integrity number (RIN), thus similar PMN time point samples were pooled (Supplemental Table 1). To ensure that the transcripts seen were those of the efferocytic cell (ST2) and not the target (hPMNs) the dataset was probed for genes unique to the target (e.g., *Ptprc, Itgam, Itgax, L-selectin*) and there was no evidence (i.e., average read count < 3) suggesting RNA contamination from the targets.

Principal Component Analysis showed that the genetic profiles of early (3 h) and late (24 h) stage efferocytic cells differ greatly from each other and from that of non-efferocytic (control) cells. (Fig. 2B). As expected, based on their functional ability to perform efferocytosis, MSCs express the transcripts for numerous phagocytic and efferocytic receptors and signaling pathways even before efferocytic challenge (Fig. 2C). Notably, phagocytic and efferocytic receptors are upregulated at 3 h post efferocytosis (*Axl, Tyro3, Itagv* etc.), while transcripts of molecules required for internal processing pathways necessary to degrade apoptotic cargo (e.g., *Elmo1, Elmo2, Dock1, Gulp1*) are upregulated at 24 h (Fig. 2C–E). Notably, MerTK, the principal receptor for efferocytosis by bone marrow macrophages, is not expressed in ST2 cells (normalized read count 25 ± 10). Therefore, MSCs demonstrate dynamic expression of key molecules in the efferocytic machinery in response to efferocytosis and a collective efferocytic machinery profile distinct from professional phagocytes within the bone marrow compartment.

While end-stage neutrophils are the most likely target in the bone marrow, we wanted to determine if MSCs are capable of engulfing other types of apoptotic cargo. We found that bone marrow-derived primary murine MSCs can also engulf apoptotic thymocytes (Fig. 2F, G). The transcriptional analysis of primary murine bone marrow-derived stromal cells exposed to apoptotic thymocytes (SCAT) identified the same efferocytic pathways (Fig. 2H–J) seen in ST2 cells, including a similar lack of MerTK expression (normalized read count 28 ± 6). These data identify the key efferocytic mediators in primary murine MSCs and suggest that similar machinery is used regardless of apoptotic target.

### Efferocytosis by MSCs induces stress response

**Fig. 3:**
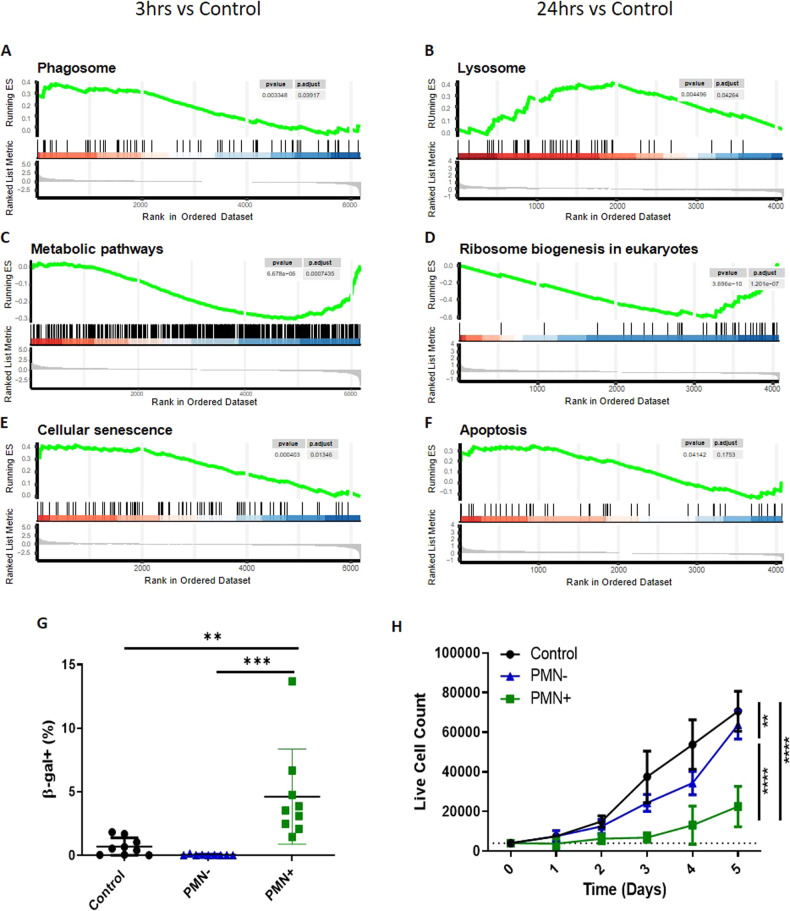
Efferocytosis by MSCs decreased expression of metabolism genes and increases stress response genes. GSEA analysis of efferocytic ST2 cell (PMN+) vs control at 3 h (**A**, **C**, **E**) and at 24 h (**B**, **D**, **F**). Gene signatures for the (**A**, **B**) processing machinery, (**C**, **D**) metabolic pathways and biogenesis, and (**E**, **F**) cellular fate. **G** ST2 cells incubated with excess (1:10) of end stage neutrophils (PMN) for 24 h and then stained for Beta-galactosidase (β-Gal) and analyzed via flow cytometry. Graphs show *N* = 9 wells (3 wells/experiment), quantified showing Mean + SD. **p < 0.01, ***p < 0.001, (ANOVA with post Tukey test). **H** ST2 cells challenged with excess neutrophils (1:10) for 24 h and then sorted alongside controls into PMN +/− and replated and counted daily over a 5-day timespan. Graphs show *N* = 4 wells, quantified showing Mean + SD. **p < 0.01, ****p < 0.0001, (ANOVA with post Tukey test).

We applied a Gene set enrichment analysis (GSEA) and pathway analysis using the Kyoto Encyclopedia of Genes and Genomes (KEGG) database (Supplemental Fig. 3) to predict the functional impact of efferocytosis on MSCs. Consistent with efferocytic behavior, there was transcriptional evidence of increased genes involved in the phagosome and lysosome (Fig. 3A, B, Supplemental Fig. 4A). In addition, this analysis identified evidence of global MSC stress through transcriptionally downregulated metabolic and biogenesis pathways (Fig. 3C, D) along with upregulated genes involved in cellular senescence and apoptosis (Fig. 3E, F). Notably, these changes occurred in both efferocytic ST2 cells and primary murine MSCs (Supplemental Fig. 4B–F) regardless of type of apoptotic cargo. To functionally confirm the increased cellular senescence identified by pathway analysis, beta-galactosidase (β-Gal) and proliferative potential were measured in efferocytic ST2 cells. β-Gal activity was increased in efferocytic (PMN+) ST2 cells compared to non-efferocytic (PMN-) ST2 cells (Fig. 3G). Consistent with increased cellular senescence, sorted PMN + ST2 cells had decreased cell replication (Fig. 3H). Therefore, our data suggest that efferocytosis induces significant cellular stress for MSCs.

### Mesenchymal stromal cell efferocytosis disrupts osteoblastic and adipocytic differentiation capacity

**Fig. 4:**
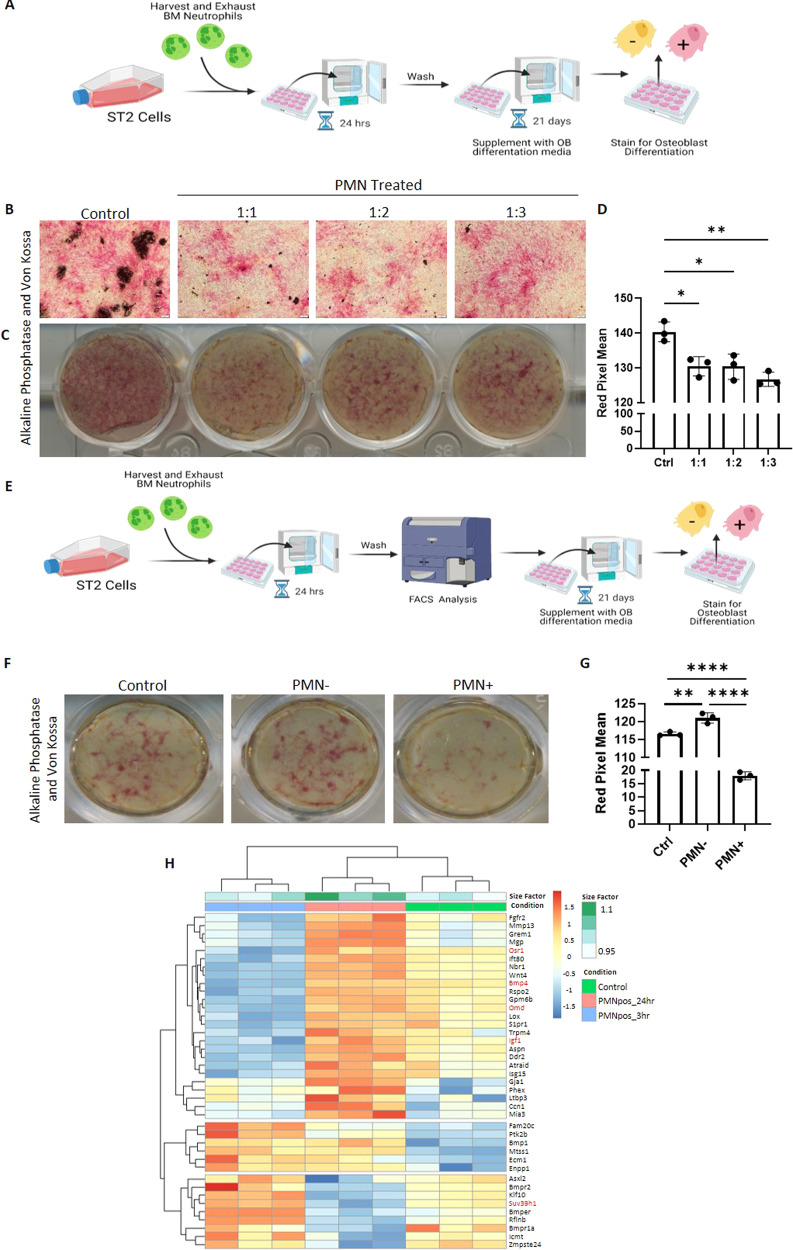
Efferocytosis of end stage neutrophils disrupts osteoblastic differentiation. **A** Experimental Schematic (**B–D**) Bone marrow-derived ST2 cells incubated with increasing concentrations of end stage neutrophils (PMN) for 24 h, then differentiated into osteoblasts using supplemented media for 21 days and stained for alkaline phosphatase (red) and Von Kossa. Representative Images of 3, quantified showing Mean+SD. *p < 0.05, **p < 0.01, (ANOVA). **E** Experimental Schematic for sorted samples (**F**, **G**) ST2 cells challenged with excess neutrophils (1:10) and then sorted alongside controls into PMN +/−, differentiated into osteoblasts using supplemented media for 21 days, and then stained for alkaline phosphatase and Von Kossa. Representative Images of 3, quantified showing Mean + SD. **p < 0.01, ****p < 0.0001, (ANOVA with post Tukey test). **H** Heat map of genes related to osteogenesis at 3 h or 24 h vs the control.

**Fig. 5:**
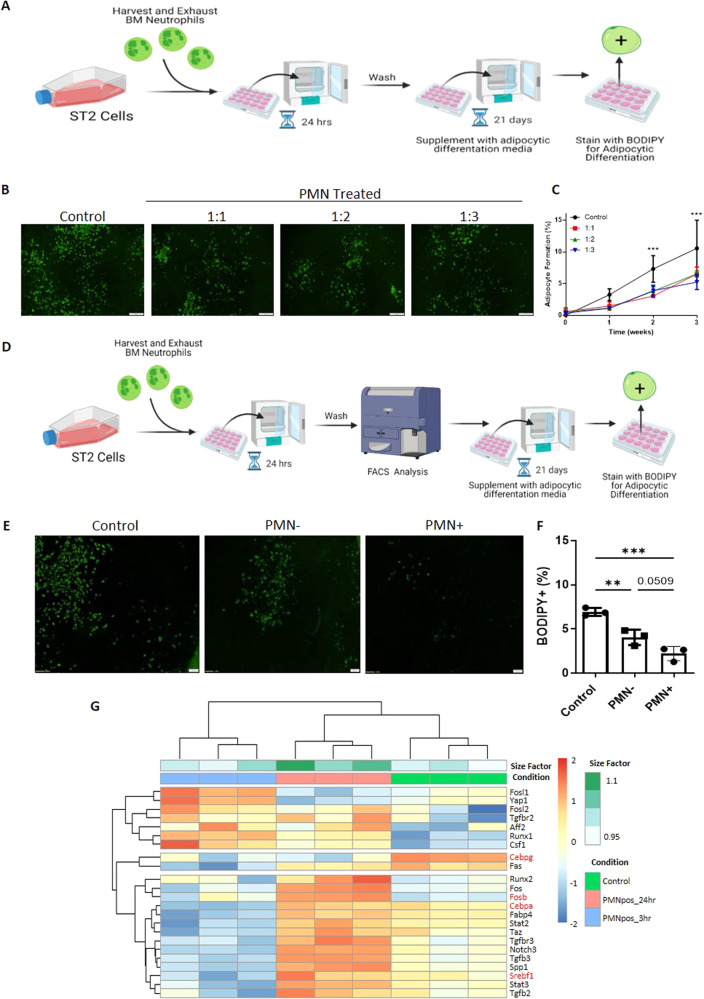
Efferocytosis of end stage neutrophils disrupts adipocytic differentiation. **A** Experimental Schematic (**B**, **C**) Bone marrow-derived ST2 cells incubated with increasing concentrations end stage neutrophils (PMN) for 24 h, then differentiated into adipocytes using supplemented media for 21 days and stained for lipid deposits with BODIPY staining. Representative images of 3, quantified showing Mean + SD. ***p < 0.001, (ANOVA). **D** Experimental Schematic for sorted samples (**E**, **F**) ST2 cells challenged with excess neutrophils (1:10) and then sorted alongside controls into PMN +/−, differentiated into adipocytes using supplemented media for 21 days, and then stained for lipid deposits with BODIPY staining. Representative Images of 3, quantified showing Mean+SD. **p < 0.01, ***p < 0.001, (ANOVA with *p*ost Tukey test). **G** Heat map of genes related to adipogenesis at 3 h or 24 h vs the control.

To test the impact of efferocytosis on MSC differentiation, we induced osteoblastic and adipocytic differentiation in MSCs following a challenge with an efferocytic target. We exposed ST2 cells to PMNs in a ST2:PMN ratio of 1:1, 1:2 and 1:3 for 24 h, and after removing non-engulfed PMNs, exposed the ST2 cells to osteoinductive or adipo-inductive media. Even though only a subset of ST2 cells were PMN+ at 24 h in prior experiments (see Fig. 1B), we found that exposure of ST2 cells to PMNs decreased osteoblastic differentiation as measured by alkaline phosphatase and Von Kossa staining in comparison to ST2 cells that were not exposed to PMNs (Fig. 4A–D). To determine if the decreased alkaline phosphatase activity was a result of cell autonomous efferocytic activity, PMN+ and PMN- ST2 cells were separated by FACS (Fig. 4E). We found that efferocytic MSCs (PMN+) had decreased alkaline phosphatase staining, while their non-efferocytic (PMN−) counterparts surprisingly had a small but significant increase in alkaline phosphatase staining in comparison to the controls, which were also subjected to the FACS fluidics without separation (Fig. 4E–G). Therefore, efferocytosis by MSCs induces cell-autonomous defects in MSC osteoblastic differentiation. Consistent with the functional defects in osteogenesis, we found that positive osteogenic regulator genes such as *Osr1, Bmp4, Omd, Igf-1*, are decreased, while negative osteogenic regulator genes such as *Suv39h1* are increased [53–55] following efferocytosis (Fig. 4H, specific genes highlighted in red). Similar to the inhibition in osteogenesis, we found that efferocytic MSCs have decreased adipocytic differentiation as shown by decreased lipid vacuole formation following induction (Fig. 5A–F). Consistent with the functional defects in adipogenesis, we found that positive adipogenic regulator genes such as *Cebpa, Cebpg, Srebf1, and Fosb* [53–55] are decreased following efferocytosis (Fig. 5G, specific genes highlighted in red). Taken together these data suggest that efferocytosis disrupts the differentiation capacity of MSCs by restraining MSC differentiation rather than by shifting from osteoblastic to adipocytic cell differentiation.

### Efferocytosis by MSCs disrupts metabolism and mitochondrial networks

**Fig. 6:**
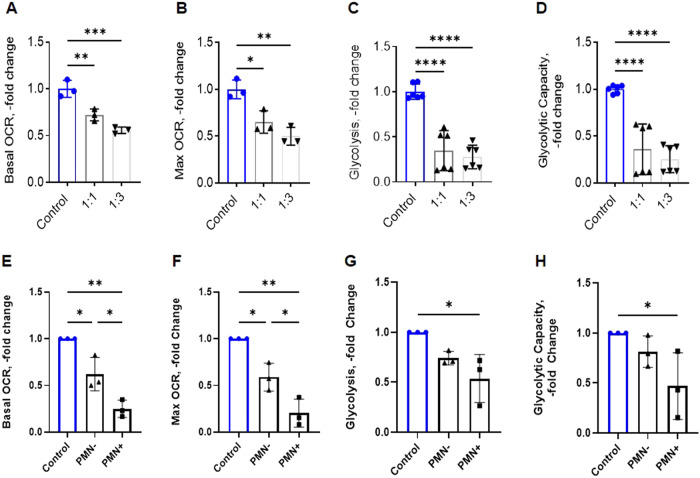
Oxidative Phosphorylation and Glycolysis disrupted in efferocytic MSCs. **A–D** Mitochondrial oxygen consumption rate (OCR), glycolysis, and glycolytic capacity measured with the Seahorse XF technology of bone marrow-derived ST2 cells incubated with increasing dosage of end stage neutrophils (PMN). Graphs show actual data points (each point contains 8 technical replicates) and calculated Mean + SD. *p < 0.05, **p < 0.01, ***p < 0.001, ****p < 0.0001 (ANOVA). **E–H** Mitochondrial oxygen consumption rate (OCR), glycolysis, and glycolytic capacity measured with the Seahorse XF technology of ST2 cells challenged with excess neutrophils (1:10) and then sorted alongside controls into PMN +/−. Graphs show biological data points (each containing 8 technical replicates) and calculated Mean + SD. *p < 0.05, **p < 0.01, (ANOVA with post Tukey test).

Many of the pathways identified in the transcriptional program of efferocytic MSCs related to dysregulation of metabolism (Fig. 3C, D). To test whether MSC efferocytosis induced metabolic disruption, we measured the oxygen consumption rates (OCR) and extracellular acidification rates (ECR), which are measurements for oxidative phosphorylation and glycolysis respectively. Consistent with our transcriptional data, we found that efferocytosis by MSCs decreases oxidative phosphorylation (Fig. 6A, B) and glycolysis (Fig. 6C, D). To determine if the decreases in oxidative phosphorylation and glycolysis were a result of environmental stress or cell-autonomous efferocytic activity in MSCs, we sorted PMN+ and PMN- MSCs and measured OCR and ECR. We found that the most drastic metabolic disruption was present in efferocytic MSCs (PMN+) for both oxygen consumption and glycolysis, while non-efferocytic (PMN−) MSCs had relatively preserved glycolysis (Fig. 6E–H). These data demonstrate that efferocytosis significantly alters metabolic processes in MSCs.

### Inhibition of mitochondrial fission decreases efferocytosis and rescues osteoblastic differentiation impairment

**Fig. 7:**
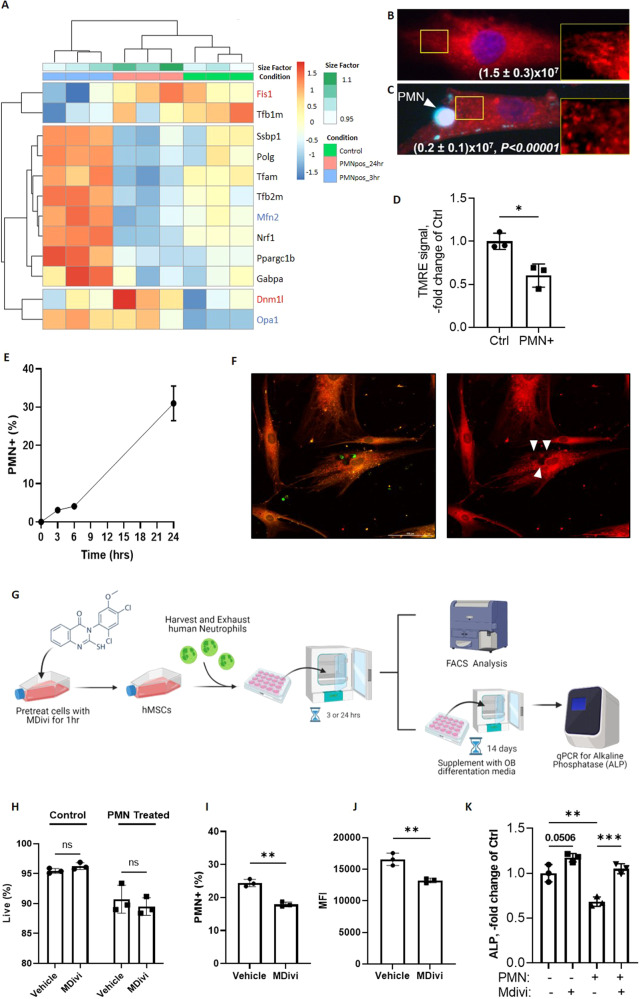
Efferocytosis shifts MSC mitochondria toward fission. **A** Heat map of mitochondrial biogenesis and dynamic genes and pathways at 3 h or 24 h vs the control in ST2 cells. Key genes regulating mitochondrial dynamics are highlighted in red (fission) and blue (fusion). **B**, **C** Representative images of control and efferocytically challenged (PMN+) hMSCs stained with MitoTracker Red (mitochondria) and DAPI (nucleus). PMN stained with Calcein AM. Highlighted regions are magnified and shown as inserts on the right of each image. ImageJ analysis of mitochondrial connectivity shown as numbers of connected pixels (Mean ± SD, *n* = 25). **D** Mitochondria from control and challenged (PMN+) hMSCs stained with tetramethyl rhodamine ethyl ester (TMRE) to measure mitochondrial membrane potential. Graphs show the biological data points *N* = 3, Mean + SD, *p < 0.05, (*t*-test). **E** Quantification of efferocytosis by hMSCs incubated excess (1:10) end stage neutrophils (hPMNs) over 24 h. *N* = 4 at each time point. Means ± SD are shown. (**F**) Representative microscopy images showing uptake of end stage neutrophil (GFP+) by hMSC (mCherry+) and the resulting voids in the cytoplasm (white arrows). **G** Experimental Schematic for samples treated with a mitochondrial inhibitor (MDivi). **H–J** Quantification via flow cytometry of viability, end stage neutrophil (PMNs) engulfment, and efficiency (mean fluorescent intensity, MFI) of hMSCs pre-treated with 25 μM Mdivi for 1 h and then given end stage PMN for 24 h. **K** Quantification of Alkaline Phosphatase (ALP) via qPCR of hMSCs pre-treated with 25 μM Mdivi for 1 h and then given end stage PMN for 3 h. *N* = 3, Mean ± SD shown on graphs **p < 0.01, ***p < 0.001, (*t*-test or ANOVA).

Since oxidative phosphorylation was impacted more heavily than glycolysis in a cell-autonomous fashion, we hypothesized that efferocytosis by MSCs may lead to mitochondrial remodeling. Using the ST2 RNA sequencing dataset, we probed for mitochondrial dynamics genes and pathways (Supplemental Fig. 5) and found efferocytosis-mediated dynamic regulation of genes associated with mitochondria fission and fusion. Early in the efferocytic process (3 h), MSCs upregulate fusion genes, such as *Mfn2* and *Opa1* (Fig. 7A, labeled in blue), however as efferocytosis progresses (24 h) MSCs upregulate fission genes, such as *Fis1* and *Dmnl1* (Fig. 7A, labeled in red). These data suggest that MSCs, after performing efferocytosis, shift from a state of mitochondrial fusion to fission. To confirm mitochondrial remodeling in efferocytic MSCs, we labelled mitochondria in control and PMN+ MSCs with MitoTracker Red or TMRE and assessed the mitochondrial length and membrane potential respectively. We found that both the mitochondrial length (Fig. 7B, C) and TMRE signal (Fig. 7D) were decreased in efferocytic MSCs, consistent with mitochondria undergoing fission. Together these data show that, as a result of efferocytosis, MSCs have diminished oxidative function and remodeled mitochondria, consistent with mitochondria undergoing fission.

Mitochondrial metabolism plays a key role in MSC ability to support tri-lineage differentiation, whereby disruption of homeostatic metabolism can impact osteoblastic differentiation and subsequent bone formation [34–36]. During normal osteoblastic differentiation MSCs undergo fusion, or mitochondrial lengthening [34, 35]. Blocking fusion and enforcing fission disrupts MSC differentiation to the osteoblastic lineage [35]. Since our data show that mitochondria undergo fission, or shortening, during MSC efferocytosis, we hypothesized that efferocytosis-induced mitochondrial fission may mediate the block in osteoblastic differentiation initiated by MSC efferocytosis. Since this is a key metabolic switch that impacts bone health and may represent a mechanism of bone loss and osteoporosis [56], we first tested if MSCs isolated from human bone marrow also are capable of efferocytosis. Human MSCs (hMSCs) demonstrated a similar rate of efferocytosis as murine MSCs (Fig. 7E, F). Similar to its effects on murine MSCs, efferocytosis also inhibited hMSCs differentiation to osteoblasts (Fig. 7K). To determine if the decreased osteoblastic differentiation potential is a result of mitochondrial fission, we treated hMSCs with Mdivi, an inhibitor for mitochondrial fission [57], prior to efferocytosis. The overall rate and efficiency (MFI) of efferocytosis, tested at 24 h, was decreased in hMSCs pre-treated with Mdivi without impacting their viability (Fig. 7G–J). Consistent with the role of mitochondrial remodeling in MSC differentiation to osteoblasts, there was a trending increase in osteoblastic differentiation with Mdivi treatment in the absence of PMN (Fig. 7K). Importantly, co-treatment with Mdivi and PMN rescued the osteoblastic differentiation potential of hMSCs (Fig. 7K). In summary, we found that inhibiting mitochondrial fission in the setting of MSC efferocytosis rescues osteoblastic differentiation potential in efferocytic MSCs. These data demonstrate that, in MSCs, increased mitochondrial fission mediates the defect in osteoblastic differentiation induced by efferocytosis.

## Discussion

Our work demonstrates that engulfment of apoptotic cells by bone marrow derived MSCs, a previously underreported function of MSCs, disrupts their metabolism and inhibits their differentiation, with potential impact for bone health. In the bone marrow microenvironment (BMME), MSCs carry out numerous functions, including the support of hematopoiesis, immunomodulation, and differentiating down the osteoblastic, adipocytic, and chondrocytic lineages to support bone growth, maintenance, and repair [58]. Previously non-professional or non-specialized phagocytes, cells that have been noted to conduct phagocytosis only under specific circumstances or only eating specific targets, were not identified in the bone marrow [3, 4]. However, MSCs had been reported to contribute to phagocytosis in the embryo [24]. Follow-up studies in vitro confirmed phagocytosis and efferocytosis capacity of MSC [16–23, 25, 26]. Given the metabolic and mitochondrial impact of efferocytosis in macrophages [6], we reasoned that MSC efferocytosis may have a metabolic impact, and that it may inhibit their differentiation.

Our data show that MSCs can act as a non-professional phagocyte in vitro using ST2 cells and primary BM MSCs from mice and humans [3, 4]. While end-stage neutrophils are the most likely targets for MSC efferocytosis, as neutrophils make up to 40–60% of the billions of cells that return to the bone marrow to be cleared [1, 52, 59], we found that MSCs can engulf other apoptotic targets, for example thymocytes, and targets from cross species (ie human PMNs by murine MSCs), suggesting that MSCs may not be selective in their efferocytic activity. This finding is consistent with the literature regarding phagocytic cells, since efferocytic receptors are known to be highly promiscuous and apoptotic engulfment signals are highly conserved even across species. Our findings on the lack of target RNA contribution of PMNs upon sequencing were not unexpected considering that it is challenging to isolate RNA even from freshly isolated non-apoptotic PMNs. Unlike other commonly used targets for efferocytic experiments such as apoptotic thymocytes, our data demonstrates that apoptotic PMNs do not contribute significant amounts of RNA to phagocytic populations even at early time points.

Transcriptomic studies of efferocytosis by MSCs found that MSCs upregulate pathways related to phagocytic behaviors, including regulation of actin cytoskeleton, focal adhesion, phagosome, and lysosome, regardless of efferocytic target type. Indeed, MSCs possess the necessary receptors to conduct efferocytosis, with Axl, Tyro3, and Megf10 being the most prominent receptors-pathways regardless of efferocytic target. In response to engulfment of apoptotic targets, MSCs also upregulate internal processing, such as Dock1, Elmo1, Gulp1, and the Axl transcriptional target and accessory protein Gas6, which is necessary to activate a functional response [60–62]. While Axl and Tyro3 are expressed, MerTK, the third TAM receptor, is not expressed on MSCs. Based on prior work, including our own, this is a key receptor pathway on professional phagocytes such as macrophages [43, 44]. These data suggest that MSCs act as a supporting phagocyte within the BMME, and they do not rely on MerTK. Since a number of small molecules have been developed to differentially target and inhibit each TAM receptor [63], it may be possible to selectively inhibit MSC efferocytosis without blocking macrophage activity.

While the most likely primary efferocytic targets for MSCs in the bone marrow are end-stage neutrophils, MSCs have also been reported to conduct phagocytosis of bacteria [16, 17], metallic particles from prosthetics [18, 19, 22], and collagen [23]. Although efferocytosis is a specialized form of phagocytosis, and the molecular mechanisms of efferocytosis closely resemble those of phagocytosis, whether phagocytosis by MSCs disrupts their metabolism and inhibits their differentiation in a cargo–dependent manner is unknown, and the mechanism for MSC phagocytosis remains to be elucidated.

The data presented here demonstrate that efferocytic activity causes a stress response in MSCs in the form of increased cellular senescence and apoptosis. As cellular senescence has been shown to decrease differentiation capacity, we hypothesized that MSC efferocytosis would decrease differentiation capacity. Consistent with this, we found that efferocytic MSCs display a diminished capacity to differentiate toward the osteoblastic and adipocytic lineages.

While cellular senescence has many causes, it has been reported to be induced by disrupted metabolic activity in which the mitochondrial dynamics between fission and fusion play a role in energy trafficking in the cell [64–66]. In addition, phagocytosis by macrophages is accompanied by changes in mitochondrial dynamics (fission) and oxidative function, which in turn promotes further phagocytosis [27]. In contrast, during MSC differentiation, mitochondria increase their fusion [34–36]. Thus, the dynamics of the mitochondrial networks between efferocytosis (fission) and differentiation (fusion) are opposing. Our RNA sequencing data found that efferocytic MSCs display decreased regulation of metabolic pathway and biogenesis genes, leading us to hypothesize that efferocytic activity in MSCs may alter mitochondrial dynamics as a mechanism that decreases their differentiation capacity. Consistent with this, we found that both oxidative phosphorylation and glycolysis, two of the major energy synthesis pathways, are decreased in MSCs following efferocytosis. Transcriptionally, the fission promoting *Fis1* and *Dmnl1* genes were upregulated. We confirmed decreased mitochondrial length and membrane potential, supporting an increase in a fission-like state for the mitochondrial network and mitochondrial dysfunction in efferocytic MSCs, suggesting that efferocytosis in MSCs impacts mitochondrial remodeling as it does in macrophages. To determine if inhibition of osteoblastic differentiation induced by efferocytosis was caused by mitochondrial remodeling, we pharmacologically inhibited fission using Mdivi [57]. Our data reinforce previous reports in which inhibition of fission increases osteoblastic differentiation independent of efferocytic activity [35], and show that pharmacologic inhibition of fission not only decreases efferocytic activity in MSCs but also rescues the defect in osteoblastic differentiation induced by MSC efferocytosis. These data demonstrate for the first time that efferocytosis impairs MSC differentiation by altering mitochondrial remodeling. While efferocytosis by MSCs may be beneficial to help professional phagocytes in the BMME by serving as a supportive non-professional phagocyte, our data suggests that this may come with detrimental consequences on osteoblastic differentiation.

Our manuscript identifies the MSCs’ role in the process of removing apoptotic cells from the bone marrow as a previously unappreciated mechanism of MSC dysfunction. Professional phagocytes, such as macrophages, regulate their non-professional counterparts, so that non-professional phagocytic cells are recruited when macrophages are either defective or insufficient to engulf apoptotic cells [3, 4, 50, 51, 67]. MSCs may therefore be engaged as non-professional phagocytic cells especially (or exclusively) when macrophage populations are depleted or dysfunctional. Consistent with this, in an embryological study on mice lacking macrophages due to genetic loss of the *pu.1* gene, MSCs gained efferocytic capabilities in vivo [24]. Relevant to the role of MSCs as skeletal precursors, Cho et al. showed that, in the absence of c-fms+ cells (early and late macrophages), there was an increase in apoptotic cells in the bone marrow, which was associated, unexpectedly, with a reduction in bone mass and bone formation [68]. In this context, it is possible that the decreased bone mass and bone loss observed may be due to recruitment and increased efferocytosis by MSCs. However in the same paper, targeting via clodronate unexpectedly did not result in bone loss [68]. The novel role of MSCs as non-professional efferocytic cells may explain this finding. While clodronate is able to target macrophages, it is non-specific, and therefore may target other phagocytic cells such as neutrophils (the main apoptotic cell population in the bone marrow), and MSCs [69]. In the setting of MSC efferocytosis, uptake of clodronate may protect the BMME by killing the MSC before it becomes senescent upon activation of efferocytic pathways, protecting from bone loss [70].

Numerous studies, including our own, have demonstrated diminished macrophage efferocytic potential and increases in apoptotic cell burden in vivo in the setting of aging and diseases such as autoimmunity, obesity and diabetes [6, 43, 68, 71, 72]. For example, we previously demonstrated significant defects in efferocytosis by bone marrow macrophages in aged mice [43], suggesting that at least some of the MSC senescence observed in aging may be a result of MSC efferocytosis. Luo et al. showed defective bone marrow macrophage efferocytosis in obese mice [72], consistent with prior reports of macrophage defects in other models of obesity and atheroschlerosis [71]. Thus, enhanced MSC efferocytosis and subsequent MSC dysfunction may represent a novel mechanism of dysfunction in bone loss and decreased bone formation associated with these conditions. In vivo studies testing MSC efferocytosis in aged and preclinical models are therefore needed.

Efferocytosis by MSCs may also have important consequences for their roles in the setting of cancer. Tumor-associated macrophages have been shown to promote tumor growth following efferocytosis, which is abundant in tumors, especially in response to cytotoxic therapies, by suppressing tumor immunity and limiting the anti-tumor response [73]. Thus, it is possible that MSCs efferocytosis could lead to an immune-suppressive/pro-tumorogenic microenvironment in bone and bone marrow in response to tumors and their metastases, especially in the setting of cytotoxic therapies. Therefore, in future studies it will be important to test the role of MSC efferocytosis as a novel mechanism of immunomodulation in the cancer microenvironment.

In this study, murine and human MSCs, which are rare populations, were by necessity removed from their microenvironment for the in vitro quantification of cell-autonomous metabolic and functional changes induced by efferocytosis. This approach, however, limits our ability to fully understand the interactions of efferocytic MSCs with other cells within the BMME, and the impact of cytokine and chemokine gradients present in vivo. Future studies are therefore needed to determine the prevalence of MSC efferocytosis in vivo during homeostasis, aging, and disease, and how other cellular and molecular components of the BMME may regulate this process.

In summary, efferocytosis by MSCs may represent a mechanism of MSC dysfunction and senescence leading to age- and disease- associated bone marrow remodeling and bone loss. Together, these data represent a novel mechanism by which MSC becomes senescent and may contribute to bone loss and disrupt the bone marrow microenvironment. Our studies also identify pharmacologically targetable mechanisms for MSC efferocytosis that may have clinical significance in the treatment of age- and disease- related bone marrow remodeling and bone loss caused, in part, by excessive MSC efferocytosis.

## Supplementary information

Supplementary Figure Legends

Figure 1E

Figure1F

Supplemental Figure 1

Supplemental Figure 2

Supplemental Table 1

Supplemental Figure 3

Supplemental Figure 4

Supplemental Figure 5
