# Supplementary material for: Efferocytosis by bone marrow mesenchymal stromal cells disrupts osteoblastic differentiation via mitochondrial remodeling
Source: Cell Death Dis. 2023 Jul 14;14(7):428. doi: 10.1038/s41419-023-05931-9 (PMC10349065; doi:10.1038/s41419-023-05931-9)
Supplement: Supplementary file 1 — Supplementary Figure Legends [file 41419_2023_5931_MOESM1_ESM.docx]

**Supplementary Video 1E:** Confocal microscopy video (middle right) captured of ST2 cells up taking an end stage neutrophil (GFP+).

**Supplementary Video 1F**: Z-stack captured of ST2 cells up taking an end stage neutrophil (GFP+).

**Supplemental Figure 1:** Representational gating strategy for quantification of engulfment of end stage neutrophils (PMN+) by ST2 cells over 24hrs.

**Supplemental Figure 2:** Representational gating scheme for sorting efferocytic ST2 cells challenged with fluorescently labeled end stage neutrophils sorted at 3 and 24hrs alongside controls for RNA sequencing.

**Supplemental Table 1:** Summary of quality check (QC) for the sorted efferocytic ST2 cells and targets. RNA concentration and RIN sources were analyzed via a NanoDropQC5.0 and Bioanalyzer QC (RNA) 5.0-Pico, respectively.

**Supplemental Figure 3:** Efferocytosis by MSCs disrupts maintenance gene pathways. KEGG analysis shows up- and down-regulation of pathways of efferocytic ST2 cells at 3hrs vs the control (A,C) and at 24hrs vs Control (B,D). KEGG analysis show up- and down-regulation of pathways of efferocytic mMSC of apoptotic thymocyte (SCAT) at 4hrs vs control (SC) (E-F).

**Supplemental Figure 4:** Efferocytosis of thymocytes by MSCs increases stress response at 4hrs. GSEA analysis of efferocytic mMSCs of apoptotic thymocytes at 4hrs SCAT vs SC. Shown are the gene signatures for (A) processing machinery, (B) biogenesis, (C-D) stress response, and (E-F) cellular fate.

**Supplemental Figure 5:** Efferocytosis of end stage neutrophils by ST2 cells disrupts metabolic activity. Heat map of genes at 3hrs or 24hrs vs the control in ST2 cells for (A) mitochondrial activity and ROS, (B) glycolytic activity, and (C) inner mitochondrial membrane.
