# Supplementary figures and images for: Efferocytosis by bone marrow mesenchymal stromal cells disrupts osteoblastic differentiation via mitochondrial remodeling
Source: Cell Death Dis. 2023 Jul 14;14(7):428. doi: 10.1038/s41419-023-05931-9 (PMC10349065; doi:10.1038/s41419-023-05931-9)

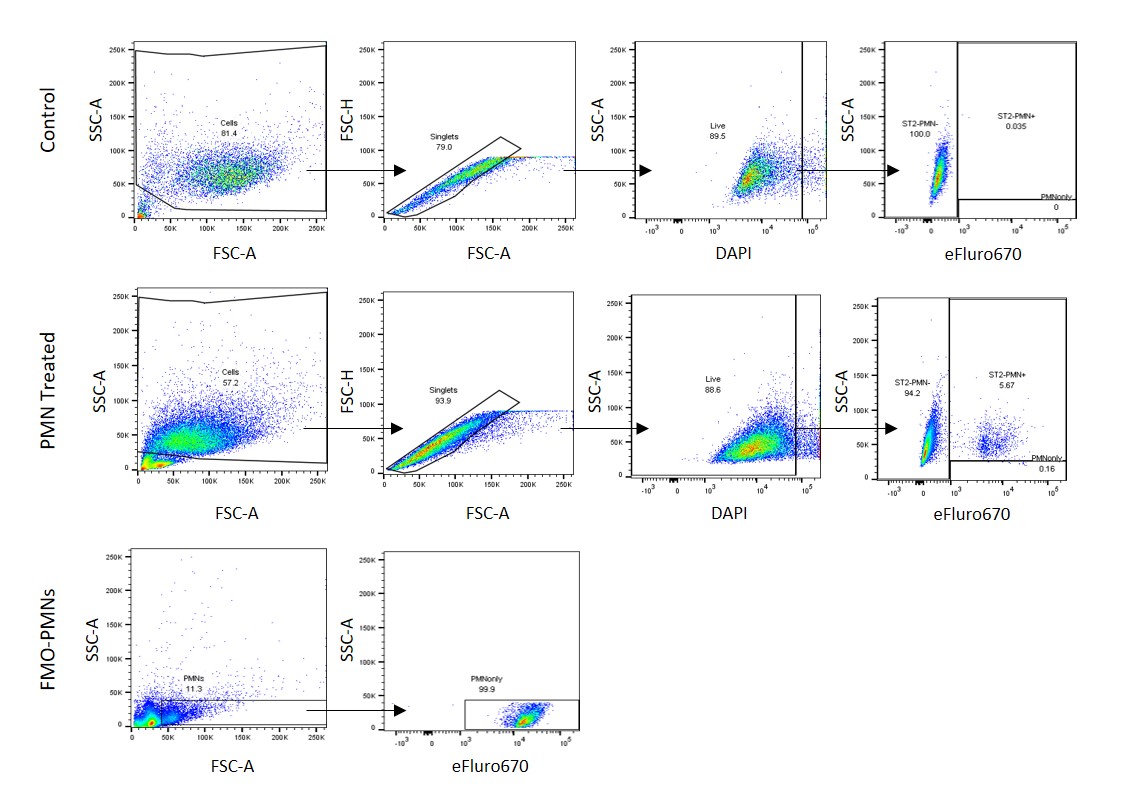

Supplement: Supplementary file 4 — Supplemental Figure 1 [file 41419_2023_5931_MOESM4_ESM.jpg]

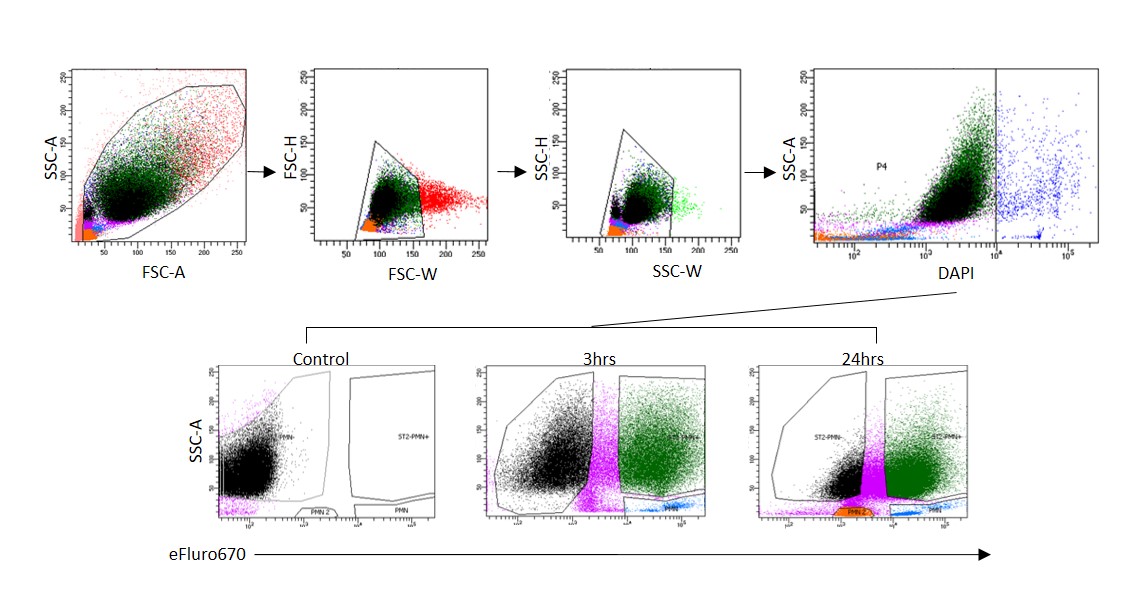

Supplement: Supplementary file 5 — Supplemental Figure 2 [file 41419_2023_5931_MOESM5_ESM.jpg]

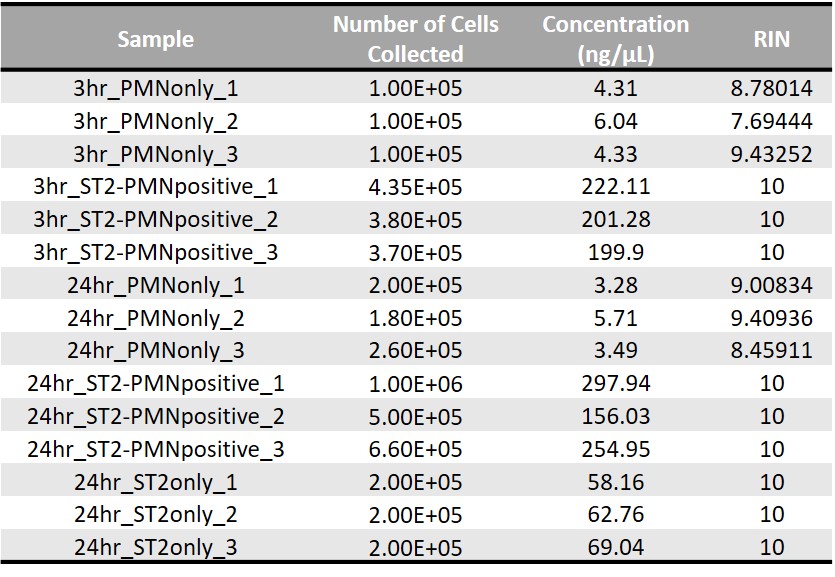

Supplement: Supplementary file 6 — Supplemental Table 1 [file 41419_2023_5931_MOESM6_ESM.jpg]

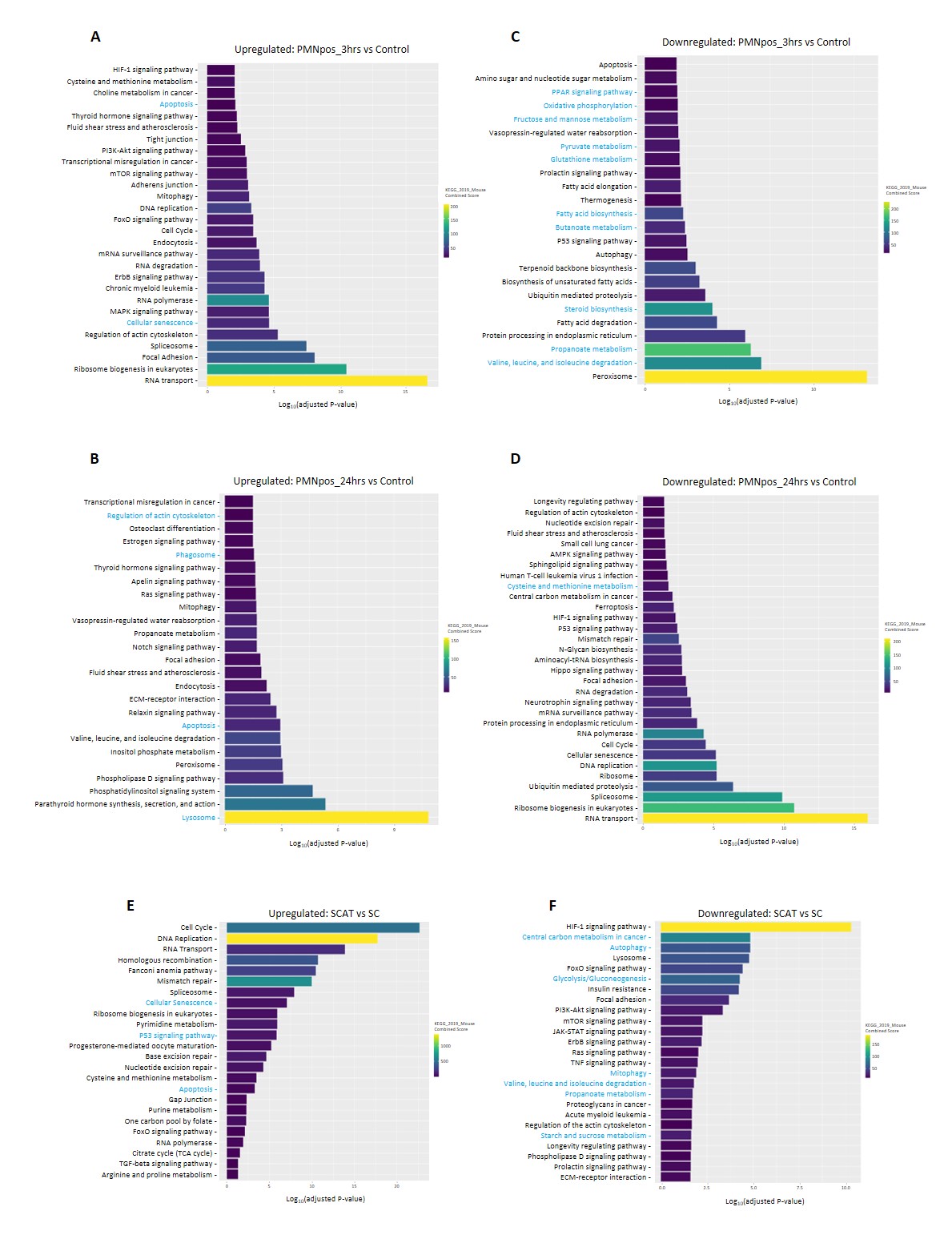

Supplement: Supplementary file 7 — Supplemental Figure 3 [file 41419_2023_5931_MOESM7_ESM.jpg]

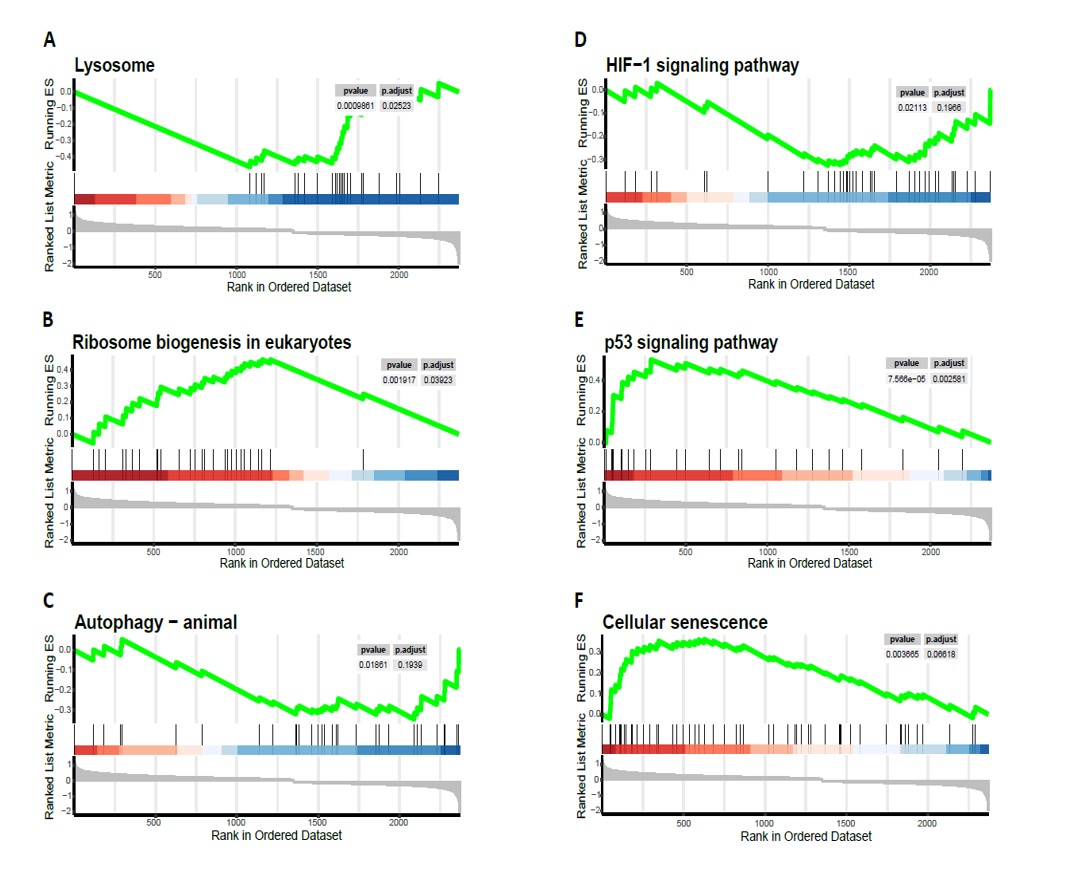

Supplement: Supplementary file 8 — Supplemental Figure 4 [file 41419_2023_5931_MOESM8_ESM.jpg]

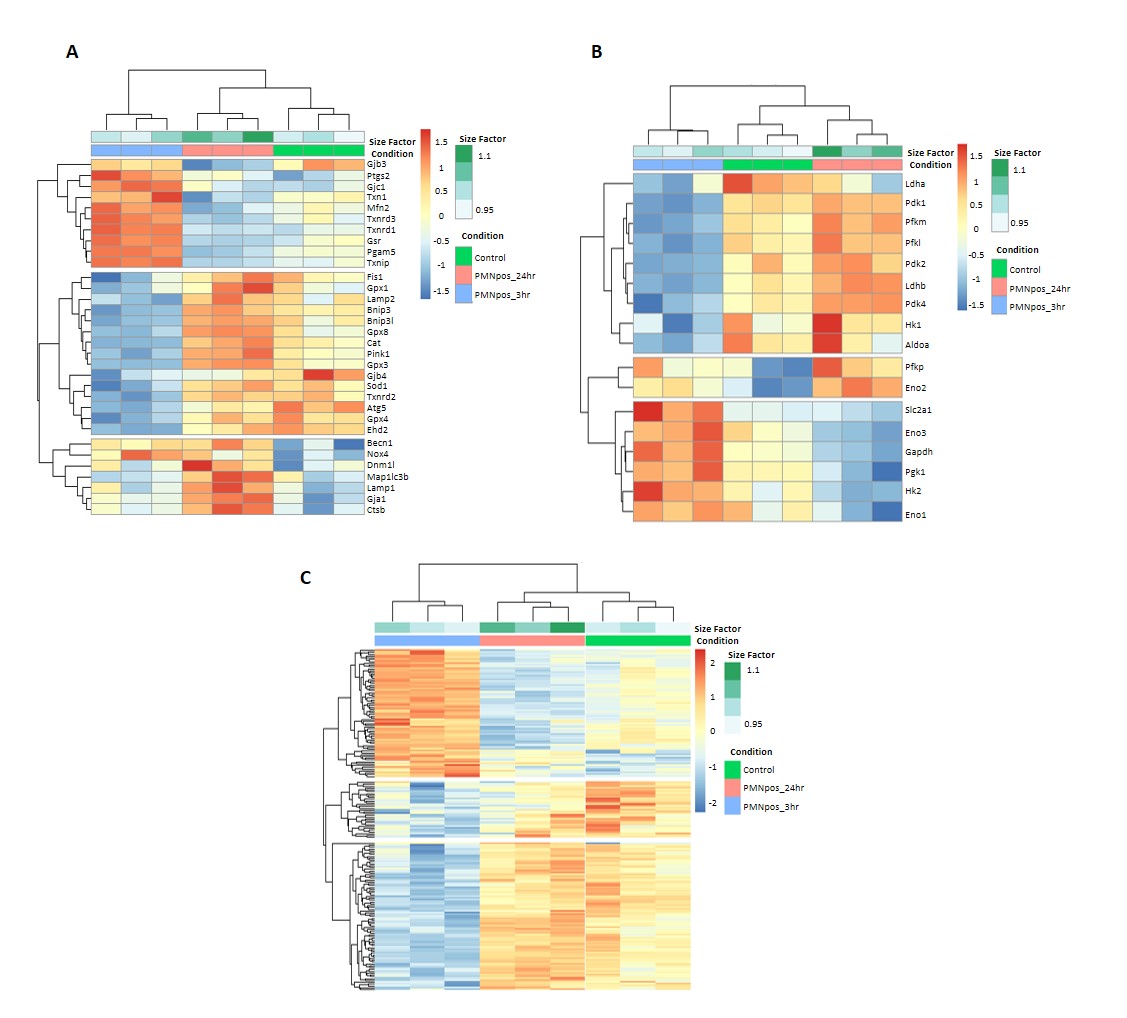

Supplement: Supplementary file 9 — Supplemental Figure 5 [file 41419_2023_5931_MOESM9_ESM.jpg]
